# Identification of an *Aeromonas hydrophila* strain as a new mosquito pathogen

**DOI:** 10.3389/fcimb.2025.1649545

**Published:** 2025-08-12

**Authors:** Rim Wehbe, Aline Karaki, Zakaria Kambris

**Affiliations:** Biology Department, Faculty of Arts and Sciences, American University of Beirut, Beirut, Lebanon

**Keywords:** mosquito pathogen, microbiota, gut damage, cell proliferation, innate immunity, *Aeromonas hydrophila*

## Abstract

The gut microbiome plays a major role in promoting organismal homeostasis. Mosquito microbiota influences various aspects of host physiology such as immunity, development and vector competence. Most studies addressing mosquito microbiota consist of microbial diversity profiling and rarely investigate the effects of individual bacteria on host physiology. This remains an important gap of knowledge, especially since not all naturally occurring gut microbes are passive commensals. Here, we identify a pathogenic strain of *Aeromonas hydrophila* that causes mortality to both *Culex pipiens* and *Aedes albopictus* mosquitoes upon ingestion. In addition, we show that *A. hydrophila* breaches the gut epithelium and gains access to the hemolymph. Parallel to gut damage, we detect a significant increase in the number of proliferative cells in the midguts of *A. hydrophila* fed mosquitoes. Moreover, we find that this bacterium induces a local immune response in the gut leading to the production of anti-microbial peptides. Finally, whole genome sequencing of the isolated strain revealed that it possesses an arsenal of virulence and resistance genes, which provides mechanistic insights into its mosquitocidal activity. This study reports a novel mosquito pathogen and highlights how a bacterial species inhabiting the gut can impact the host’s survival and homeostasis.

## Introduction

The insect gut is known to be inhabited by a simple microbiota consisting of beneficial and harmless microorganisms (Broderick and Lemaitre, 2012). Recent studies have shown that the microbiota influences nearly all aspects of host physiology, and that the gut microbiome plays a major role in promoting overall organismal homeostasis (Lesperance and Broderick, 2020). Primary work done in *Drosophila melanogaster* has shown that even a low-diversity microbiota can highly impact the host development and metabolic functions (Shin et al., 2011). In fact, upon nutrient shortage, *Lactobacillus plantarum*, a common *D. melanogaster* gut bacterium, was sufficient on its own to mimic the function of the natural microbiota by regulating hormonal growth signaling when introduced into axenic larvae (Storelli et al., 2011). Results obtained in *D. melanogaster* paved the way for studies investigating the role of microbiota in disease vectors, particularly in mosquitoes. Investigations on the microbial communities constituting the gut microbiota identified some “core” bacterial taxa that are host specific. For example, Saab et al. (2020) showed that when *Anopheles gambiae* and *Aedes albopictus* were co-cultured in the same laboratory conditions, their gut microbiota remained distinct. Other studies argued that the gut microbiota, in its majority, is transient rather than resident and varies according to extrinsic factors, mainly diet (Kang et al., 2020; Chandler et al., 2011).

The mosquito’s gut plays a pivotal role in many physiological functions such as food digestion, immune surveillance and local immunity. In fact, the gut acts as a physical barrier preventing ingested bacteria from reaching the hemocoel. Additionally, it is considered the primary site of pathogen interaction, as well as microbiota housing (Gabrieli et al., 2021; Caragata et al., 2019). The interplay between host immunity and microbial homeostasis is mediated by the Toll, Imd, and JAK-STAT pathways (Yu et al., 2022; Garver et al., 2012). In systemic immune response, both Imd and Toll signaling pathways are involved in controlling the expression of different anti-microbial peptides (AMPs) (Hixson et al., 2024). However, in gut immunity, the local production of AMPs is dependent only on the Imd signaling pathway (Lemaitre and Hoffmann, 2007). The Imd signaling pathway is activated by diaminopimelic acid (DAP)-type peptidoglycan (PGN) of Gram-negative bacteria via the PGRP-LC receptor. Consequently, the downstream nuclear transcription factor kappaB (NF-kB) (Relish in *D. melanogaster*, Rel2 in mosquitoes) is transferred to the nucleus, where it induces the expression of AMP-encoding genes, including diptericin (in *D. melanogaster*) and cecropins (in both *D. melanogaster* and mosquitoes) (Leulier et al., 2003; Kumar et al., 2018). Studies focusing on the role of the microbiota in mosquitoes found that the natural gut bacteria may affect vector competence, as they have the potential to “prime” the immune system leading to better host protection upon later exposure to pathogens (Ramirez et al., 2012; Dong et al., 2009; Bai et al., 2019). Other studies showed that gut-resident bacteria might alter the gut environment and inhibit its defenses by influencing pathogen attachment or replication (Wu et al., 2019).

Unlike in *D. melanogaster* (Buchon et al., 2013; Buchon and Osman, 2015), one less studied aspect of mosquito physiology is stem cell regeneration. Janeh et al. (2017) provided the first evidence for the presence of regenerative cells in adult *A. albopictus* midguts and showed that these cells proliferate in the midgut after the ingestion of pathogenic bacteria or following chemical damage. Similar findings were observed in adult *Culex pipiens* mosquitoes (Janeh et al., 2019). Furthermore, Taracena et al. (2024) showed that dividing cells were also present in adult *An. gambiae* midguts, and their occurrence increased upon blood feeding. Other recent work demonstrated that midgut cell proliferation could also be regulated by hemocytes (Cardoso-Jaime and Dimopoulos, 2025). Additionally, it has been reported that intestinal stem cell (ISC) renewal and midgut integrity influence the mosquito’s ability to transmit viruses (Taracena et al., 2018; Hixson et al., 2021). All these findings highlight the importance of the gut, not only as a passive physical barrier but also as a highly plastic structure that can be affected by many factors such as host species, the environment, and microbial interactions.

Despite the growing body of literature focusing on the importance of the microbiota on host physiology, most studies of mosquito microbiota remain descriptive. These studies primarily consist of microbial diversity profiling and metagenomics surveys, without directly addressing the effects of individual bacteria on host physiology. This represents a significant knowledge gap, especially since not all naturally occurring gut microbes are passive commensals. In fact, some might act as symbionts, while others can act as opportunistic pathogens, having negative effects on the host (Wu et al., 2019; Nehme et al., 2007). In this context, we observed an unusual increase in larval mortality in the laboratory reared *C. pipiens*, which coincided with the introduction of new larvae/water to the insectary. We isolated a strain of *Aeromonas hydrophila* (*A. hydrophila*) from the guts of dying *C. pipiens* larvae and determined that it was responsible for the observed mortality. *A. hydrophila* is a well-documented pathogen in mammals, birds, and fish, causing a range of diseases from tissue necrosis to septicemia (Semwal et al., 2023). *A. hydrophila* has also been reported in several mosquito microbiota-profiling studies (Silva et al., 2021; Darbandsari et al., 2025; Rodpai et al., 2023; Li et al., 2024). However, the effects of this bacterium on disease vectors, especially on adult mosquitoes, remain unknown. In this study, we showed that the new *A. hydrophila* strain is a virulent mosquito pathogen, capable of killing both *C. pipiens* and *A. albopictus* mosquitoes upon ingestion. In addition, our results indicated that *A. hydrophila* can severely damage the gut, allowing access to the hemolymph. Consistent with observed gut damage, we detected a significant increase in the number of proliferative cells in the midguts. Moreover, we found that feeding on *A. hydrophila* elicits a localized immune response within the gut epithelium, leading to the production of AMPs via the Imd pathway. Finally, complete genome sequencing of the isolated *A. hydrophila* strain revealed that it possesses an arsenal of virulence factor genes, providing insights into the possible mechanisms by which this strain exerts its mosquitocidal activity.

## Materials and methods

### Mosquito and *D. melanogaster* strains and rearing

All animal procedures were carried according to protocols approved by the Institutional Animal Care and Use Committee (IACUC) at the American University of Beirut (AUB), and all methods were carried out in accordance with relevant IACUC guidelines and regulations. Adults were continuously supplied with cotton pads soaked in a 10% sucrose solution and had access to water cups containing clean tap water. Larvae were fed on yeast for the first 24 hours, then on fish pellet food till pupation. Pupae were collected with a plastic pipette and placed in water cups inside plastic cages. A local strain of *Aedes albopictus* mosquitoes (originally captured from Sarba in the suburbs of Beirut, Lebanon) was maintained in the insectary at 28°C and 75% humidity using a 12:12 light/dark photocycle. This strain has been kept in the insectary for more than 10 years. Feeding was allowed on anesthetized mice, and eggs were collected on filter paper four days after the blood meal. Eggs were dried for two weeks before inducing hatching by immersion in aged tap water. A local strain of *Culex pipiens* mosquitoes (Makhoul strain captured from the AUB neighborhood, Beirut, Lebanon) was used in this study. This strain has been kept in the insectary since 2014. Egg rafts were collected once every generation and were allowed to hatch in tap water. *D. melanogaster W^1118^* strain and *Relish^E20^* were used in these experiments. Stocks were reared in 50 mL vials containing standard cornmeal agar food, prepared according to the *D. melanogaster* Bloomington Stock Center recipe. The main stocks were kept at 18°C, with the humidity set at 45%, under a 12:12 light/dark photocycle.

### Bacterial strains

The bacterial strains used in this study were *Serratia marcescens* pGEN222, ampicillin-resistant and GFP labelled *Escherichia coli*, and the isolated *Aeromonas hydrophila* strain. Bacterial strains were cultured overnight in Luria-Bertani (LB) broth at 33°C with shaking at 180 rpm. The overnight cultures were centrifuged at 5,000 × g for 10 minutes, and the bacterial pellets were washed once in sterile phosphate-buffered saline (PBS, pH 7.4). The washed pellets were resuspended in PBS to obtain an optical density (OD_600_) of 1. Serial 10-fold dilutions were then prepared, and 100 µL from appropriate dilutions were plated onto LB agar plates containing ampicillin, and colony-forming units (CFU) were counted to calculate the CFU/mL in the original suspension. All experiments were performed using the same protocol for both bacterial species.

### Bacterial treatments

For infection experiments, mosquitoes and *Drosophila* were aged between 5 and 7 days old. Flies were starved for 2 hours before their cups were supplemented with cotton pads soaked in 10% sucrose (for controls), or a bacterial suspension OD600 = 15. Flies were allowed to feed continuously until the guts were dissected for immunohistochemistry and Real-Time PCR, 24 hours after the start of treatment.

### Survival assays

Female mosquitoes and *Drosophila* were starved for 2 hours before their cups were supplemented with cotton pads soaked in 3% sucrose for controls, or in a bacterial suspension (OD600 = 15) in 3% sucrose. Dead insects were counted at different time intervals. Each infection was done in triplicate with 15–20 females for each mosquito species and *Drosophila* per experiment, and the rates of survivals were plotted as function of time. For statistical analysis of the survival data, the log-rank (Mantel-Cox) test was performed.

### Isolation of mosquito and *D. melanogaster* midguts

Flies were cold anesthetized by placing the cups on ice, then transferred one at a time onto a glass slide in a drop of 1X PBS. The isolation of midguts was performed under a light stereomicroscope. Using fine forceps, the animal head was cut, and the mosquito abdomen was pulled from the posterior end until the midgut detaches. The isolated midguts were then placed in 1.5 ml Eppendorf tubes containing 1X PBS and kept on ice.

### Fixation and staining

Isolated guts were fixed for 30 minutes using a 4% Paraformaldehyde (VWR, USA) solution in 1X PBS. This was followed by three 15-minute washes in PBS-Triton 0.1% to allow permeabilization of the guts. Blocking was then performed for 30 minutes by adding a solution of 1X PBS -Triton 0.1%-BSA 2%. After blocking, the primary rabbit α -PH3 antibodies (ABCAM, UK) were added (1:800 in 1X PBS-Triton 0.1%-BSA 2%) overnight at 4°C. Following another three 15-minute washes in PBS-Triton 0.1%, the secondary antibodies Alexa Fluor^®^ 594 (ABCAM, UK) were added (1:800 in PBS-Triton 0.1%-BSA 2%) for 3 hours at room temperature. After secondary antibodies removal, DAPI stain was applied at a concentration of 1:10000 for 2 minutes. Three final washes in PBS-Triton 0.1% were performed before mounting the guts on microscope slides in anti-fade medium (Immu-Mount, Thermo Scientific). Finally, coverslips were sealed with colorless nail varnish.

### Fluorescent microscopy, cell counting and statistical analysis

The slides prepared were observed under an inverted fluorescence microscope (Zeiss Axiovert 200, Source: AttoArc2 HBO 100 W) for counting proliferating cells, and an upright fluorescence microscope (Leica DM6 B) for image acquisition. Cell counts were analyzed using the Graphpad Prism software, and an unpaired *t* test was performed.

### CFU assays

Mosquitoes were starved for 2 hours before being placed on sucrose alone (control) or sucrose supplemented with a suspension of ampicillin-resistant *E. coli* GFP (OD600 = 50) or *A. hydrophila* (OD600 = 15), for 24 hours. The bacterial suspension was then replaced by sucrose and the hemolymph from anesthetized mosquitoes (after clipping their proboscis) was collected into 1xPBS, 24 hours later. For mosquitoes co-infected with both *A. hydrophila* (OD600 = 15) and *E. coli GFP* (OD600 = 50), they were starved for 2 hours before being placed on sucrose supplemented with both bacteria: *A. albopictus* for 24 hours and *C. pipiens* for 36 hours. Finally, the mosquitoes were placed on sucrose solutions for another 12 hours. Dilutions in sterile LB of approximately 5 μl of hemolymph were plated on LB plates supplemented with ampicillin (100 μg/mL). The colonies were counted to estimate the approximate CFUs per mosquito.

### Real-time PCR

Dissected guts were directly placed and homogenized in TRIzol^®^. RNA was extracted using chloroform and precipitated with isopropanol according to the manufacturer’s instructions (Invitrogen). The extracted RNAs were quantified using a nanodrop spectrophotometer (Thermo), and 500 ng were retrotranscribed into cDNA (iScript Biorad) for each sample. Real-time PCR was performed in the presence of SYBR green (Qiagen) on 1/20 dilutions of the RT reactions, using a BIO RAD thermocycler (CFX 96 Real-timeSystem, C1000). Ct values for target genes were normalized to Rsp7 and compared to controls using the delta Ct method. A minimum of three independent experiments were averaged and unpaired t tests were performed. Primers were designed using the Primer3 online software.

*A. albopictus*:

Rp49 Forward: 5’-AGTCGGACCGCTATGACAAG-3’
Rp49 Reverse: 5’-GACGTTGTGGACCAGGAACT-3’
Cecropin A1 Forward: 5’-GAGTCGGCAAACGAGTCTTC-3’
Cecropin A1 Reverse: 5’-TTGAACCCGGACCATAAATC-3’

*C. pipiens:*

Rp49 Forward: 5’-AAGAAGCGCAAGCTGATTGT-3’
Rp49 Reverse: 5’-CGACGGGTAATCGAATTTGT-3’
Cecropin A2 Forward: 5’-TTGCAATTGTCCTGTTGGCC-3’
Cecropin A2 Reverse: 5’-AGTGCATTAATTCCAGCAACCA-3’

*D. melanogaster:*

Rpl32 Forward: 5’-GACGCTTCAAGGGACAGTATCTG-3’
Rpl32 Reverse: 5’-AAACGCGGTTCTGCATGAG-3’
Diptericin Forward: 5’-GCTGCGCAATCGCTTCTACT-3’
Diptericin Reverse: 5’-TGGTGGAGTGGGCTTCATG-3’

### Bacterial genomic DNA extraction

Genomic DNA was extracted from the bacterial isolate cultured on Luria Bertani agar using the Quick-DNA™ Fungal/Bacterial Miniprep kit (Zymo Research, Irvine, CA). Cells were lysed by mechanical disruption in ZR BashingBead™ lysis tubes. DNA was purified using spin-column technology and the Genomic DNA Clean & Concentrator™ kit (Zymo Research) according to the manufacturer’s protocols. NanoPhotometer N60 spectrophotometer (Implen, Munich, Germany) was used for the quantification of purified DNA.

### Short-read whole-genome sequencing – Illumina

DNA library preparation was performed using the Illumina DNA prep kit (Illumina, San Diego, CA), according to Illumina’s protocol: DNA underwent an initial tagmentation step followed by post-tagmentation cleanup. Then, tagmented DNA was amplified with addition of DNA/RNA unique dual (UD) indexes (Illumina) followed by library cleanup and elution. The concentration of DNA libraries were measured using the Qubit dsDNA High Sensitivity assay kit (Invitrogen, Waltham, MA) on a Qubit 4 fluorometer (Invitrogen). DNA libraries were pooled, denatured and diluted to 12 pM. PhiX Control v3 (Illumina) was added then the pooled library was sequenced using a MiSeq V2 Reagent Kit (500 cycles) on an Illumina MiSeq platform (Illumina) for 250×2 cycles and achieved a 50x coverage.

### Long-read whole-genome sequencing – Oxford Nanopore Technologies

DNA library preparation was performed using the rapid barcoding kit (V14) (ONT, Oxford, UK) according to ONT’s protocol. Briefly, bacterial genomic DNA underwent barcoding, pooling then cleanup. Then, the resulting DNA libraries were eluted and their concentrations were measured on a Qubit 4 fluorometer (Invitrogen). Finally, the DNA libraries were loaded on an R10.4.1 flow cell (ONT) and sequenced on an Mk1B device (ONT).

### Bioinformatics analysis

Raw short and long reads were used to reconstruct the genome using Unicycler assembler (hybrid assembly) (PMID: 28594827). QUAST (PMID: 23422339) was used to assess the quality of the assembled genome and to extract the percentage of GC-content and the total length of the genome. To confirm the isolate species, KmerFinder tool (PMID: 24172157) was applied on the FASTA assembled genome. Antimicrobial resistance genes were identified using the Comprehensive Antibiotic Resistance Database (CARD) (PMID: 36263822). Genome annotation was performed using PROKKA annotation tool (PMID: 24642063). Circos plot was generated by converting the PROKKA GenBank file to XML format and plotted using cgview tool (PMID: 15479716). The genome data for *A. hydrophila* has these references (Bioproject number: PRJNA613441 - BioSample: SAMN48681286 - SRA: SRS25118285) and can be accessed via the following link: https://www.ncbi.nlm.nih.gov/biosample/48681286.

## Results

### *A. hydrophila* is a potent pathogen to both *C. pipiens* and *A. albopictus* mosquitoes

**Figure 1:**
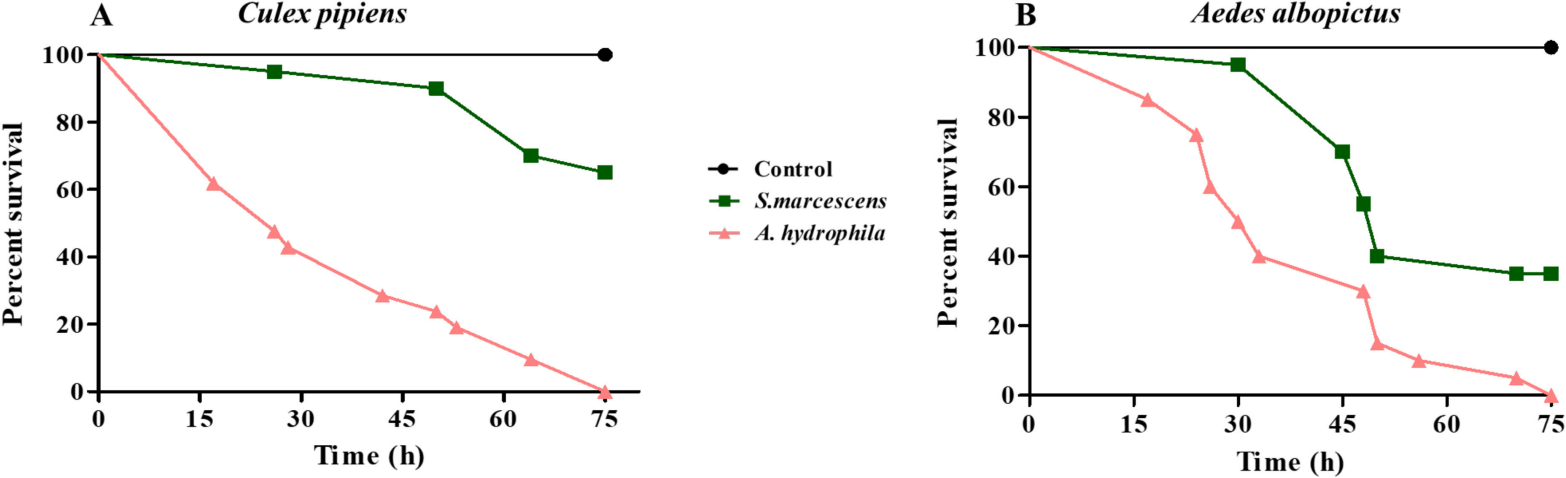
*A. hydrophila* is a potent pathogen to both *C. pipiens* and *A. albopictus* mosquitoes. Survival of *C. pipiens* **(A)** and *A. albopictus* **(B)** after feeding on sucrose solutions (control) or sucrose supplemented with *S. marcescens* (OD600 = 15) or *A. hydrophila* (OD600 = 15). *A. hydrophila* caused significant death in *C. pipiens* and *A. albopictus*. The killing effect was stronger than that of *S. marcescens* in both mosquito species. Survivals rates were plotted as a function of time. Within each panel, all survival curves had statistically significant differences (p < 0.001). One representative graph is shown for each mosquito species. The experiments were done in triplicate with a minimum of 15 females for each mosquito species per experiment.

An unusual increase in larval death was observed in the laboratory reared *C. pipiens* colony. This coincided with the introduction of new larvae/water to the insectary. We suspected that this mortality could be due to a bacterial infection. For this reason, we took the guts of *C. pipiens* dying larvae and plated them on LB plates. Interestingly, one phenotypically distinct cultivable bacterium was highly predominant. 16S rRNA gene sequencing revealed that the isolated bacterium was *Aeromonas hydrophila*. This Gram-negative bacterial strain was resistant to several antibiotics including ampicillin (**Table 1**). To determine whether *A. hydrophila* was responsible for *C. pipiens* mortality, adult female mosquitoes were starved before being allowed to feed on either sucrose solution (control), sucrose supplemented with *Serratia marcescens*, a Gram-negative bacteria used as a model to infect insects (Nehme et al., 2007), or sucrose supplemented with *A. hydrophila* (OD600 = 15 which corresponded approximately to 2.4 x 10^15^ CFU/mL for *S. marcescens* and to 1.05x10^12^ CFU/mL for *A. hydrophila*). Mosquito survival was monitored and the results showed that feeding on *A. hydrophila* caused high mortality in *C. pipiens*, where approximately 50% of the mosquitoes were killed 30 hours post-infection (**Figure 1A**). This mortality was significantly higher than that caused by *S. marcescens*. To check whether *A. hydrophila* is also pathogenic to other disease vectors, the same experiment was performed on *Aedes albopictus* mosquitoes. The survival analysis showed that 50% of *A. albopictus* fed on *A. hydrophila* died only 24 hours post-infection, which is faster than the killing effect observed in *C. pipiens* (**Figures 1A, B**). In addition, as observed in *C. pipiens*, *A. hydrophila* killed *A. albopictus* at a higher rate than *S. marcescens.* These results indicate that *A. hydrophila* is a new mosquito pathogen.

**Table 1:** List of main genes associated with virulence and antibiotic resistance found in *A. hydrophila* genome.

Virulence/Pathogenicity		Antibiotic resistance	
Gene	Product	Gene	Product
*hlyA*	Hemolysin	*cphA*	Metallo-beta-lactamase type 2
*hlyD_1, hlyD_2*	Hemolysin secretion protein D, chromosomal	*bla*	Beta-lactamase OXA-18
*HlyB*	Alpha-hemolysin translocation ATP-binding protein	*ampC*	Beta-lactamase
*aerA_2*	Aerolysin	*bmr3*	Multidrug resistance protein 3
*apxIB_2*	Toxin RTX-I translocation ATP-binding protein	*mdtH*	Multidrug resistance protein MdtH
*eta*	Exotoxin A	*mdtL_2*	Multidrug resistance protein MdtL
	Virulence protein	*mdtA_3*	Multidrug resistance protein MdtA
*phoQ*	Virulence sensor histidine kinase PhoQ	*mepA_1*	Multidrug export protein MepA
*sctC_1*, *sctC_2*	Type 3 secretion system secretin	*mdtN*	Multidrug resistance protein MdtN
*ecpD*	Fimbria adhesin EcpD	*norM_1*	Multidrug resistance protein NorM
*tabA*	Toxin-antitoxin biofilm protein TabA	*mdtE*	Multidrug resistance protein MdtE
*phoE_1*	Outer membrane porin PhoE	*emrD*	Multidrug resistance protein D
*ompC*	Outer membrane porin C	*acrB*	Multidrug efflux pump subunit AcrB
*chiP*	Chitoporin	*acrA*	Multidrug efflux pump subunit AcrA
*tdeA*	Toxin and drug export protein A	*acrE*	Multidrug export protein AcrE
		*mdtC*	Multidrug resistance protein MdtC
		*mdlB*	Multidrug resistance-like ATP-binding protein MdlB
		*macA_1*	Macrolide export protein MacA
		*macB_1*	Macrolide export ATP-binding/permease protein MacB

### Feeding on *A. hydrophila* significantly increases the number of mitotic cells in the midguts of *C. pipiens* and *A. albopictus*

**Figure 2:**
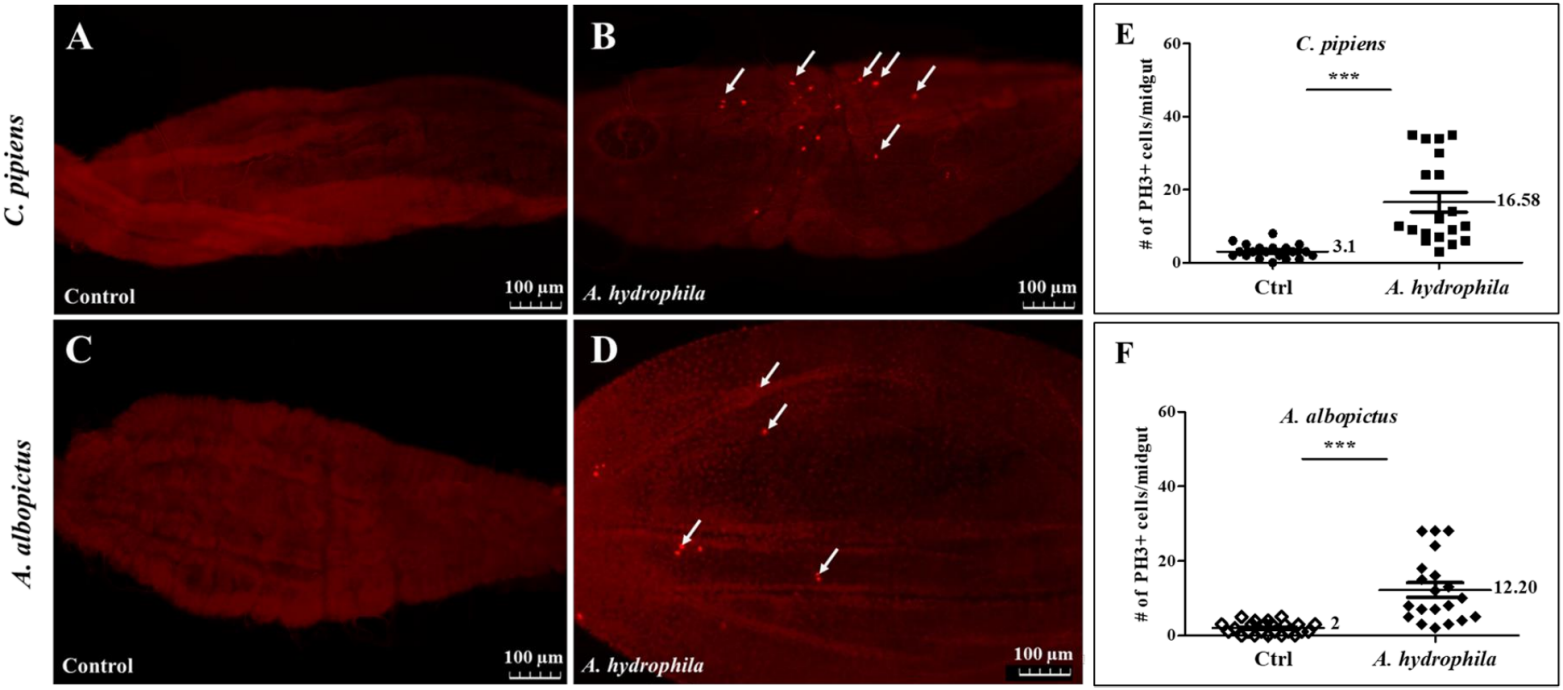
Feeding on *A. hydrophila* significantly increases the number of mitotic cells in the midguts of *C. pipiens* and *A. albopictus*. Anti-PH3 antibodies labelling shows that replicative cells increase 24 hours after feeding on *A. hydrophila* (OD600 = 15) in both *C. pipiens* **(B)** *and A. albopictus* **(D)** midguts in comparison to non-infected controls **(A, C)**. Arrows point to representative PH3-positive cells. Quantification of these results show that the increase in the number of replicative cells at the level of the midguts is significant in both mosquito species (p < 0.0001). In *A. hydrophila* fed *C. pipiens* mosquitoes, an average of 16.58 ± 2.711 PH3 positive cell per midgut (n=19) was observed as compared to the midguts of sucrose fed mosquito **(E)** that showed an average of 3.100 ± 0.4286 (n=20). *A. albopictus* mosquitoes exhibited a similar response **(F)** 12.20 ± 1.976 dividing cell per midgut (n=20) was detected when compared to controls that had 2.000 ± 0.3297 dividing cell per midgut (n=24).

We have previously shown that dividing cells exist in the midguts of both *C. pipiens* and *A. albopictus*, and that the number of these cells significantly increases following chemical or bacterial damage (Janeh et al., 2017, 2019). Taking into consideration that *A. hydrophila* causes death upon oral ingestion, we speculated that this bacterium is able to cause gut damage and induce cell division. To explore this possibility, adult females of the two mosquito species were starved before feeding on either sucrose (control), or sucrose supplemented with *A. hydrophila* (OD600 = 15). Mosquito guts were dissected 24 hours post-feeding and stained using anti-phospho-histone H3 protein antibodies (anti-PH3), a specific marker of mitotic cells (Taracena et al., 2018). In both *C. pipiens* and *A. albopictus* midguts, an increase in the number of dividing cells was observed after feeding on *A. hydrophila* (**Figures 2B–D**), in comparison to the control groups (**Figures 2A, C**). The quantification of the proliferating cells revealed statistically significant differences, as shown in (**Figures 2E, F**). Indeed, in *A. hydrophila* fed *C. pipiens* mosquitoes, an average of 16.58 ± 2.711 PH3 positive cells per midgut (n=19) was observed, as compared to the midguts of sucrose-fed mosquitoes, which showed an average of 3.100 ± 0.4286 (n=20) (E). Similarly, in *A. albopictus*,12.20 ± 1.976 dividing cells per midgut (n=20) were detected, when compared to controls which showed 2.000 ± 0.3297 dividing cells per midgut (n=24) (F). This implies that *A. hydrophila* damages the midgut, and that increased cell proliferation aims to repair the damage.

### Ingestion of *A. hydrophila* leads to leaky guts in both *C. pipiens* and *A. albopictus*

**Figure 3:**
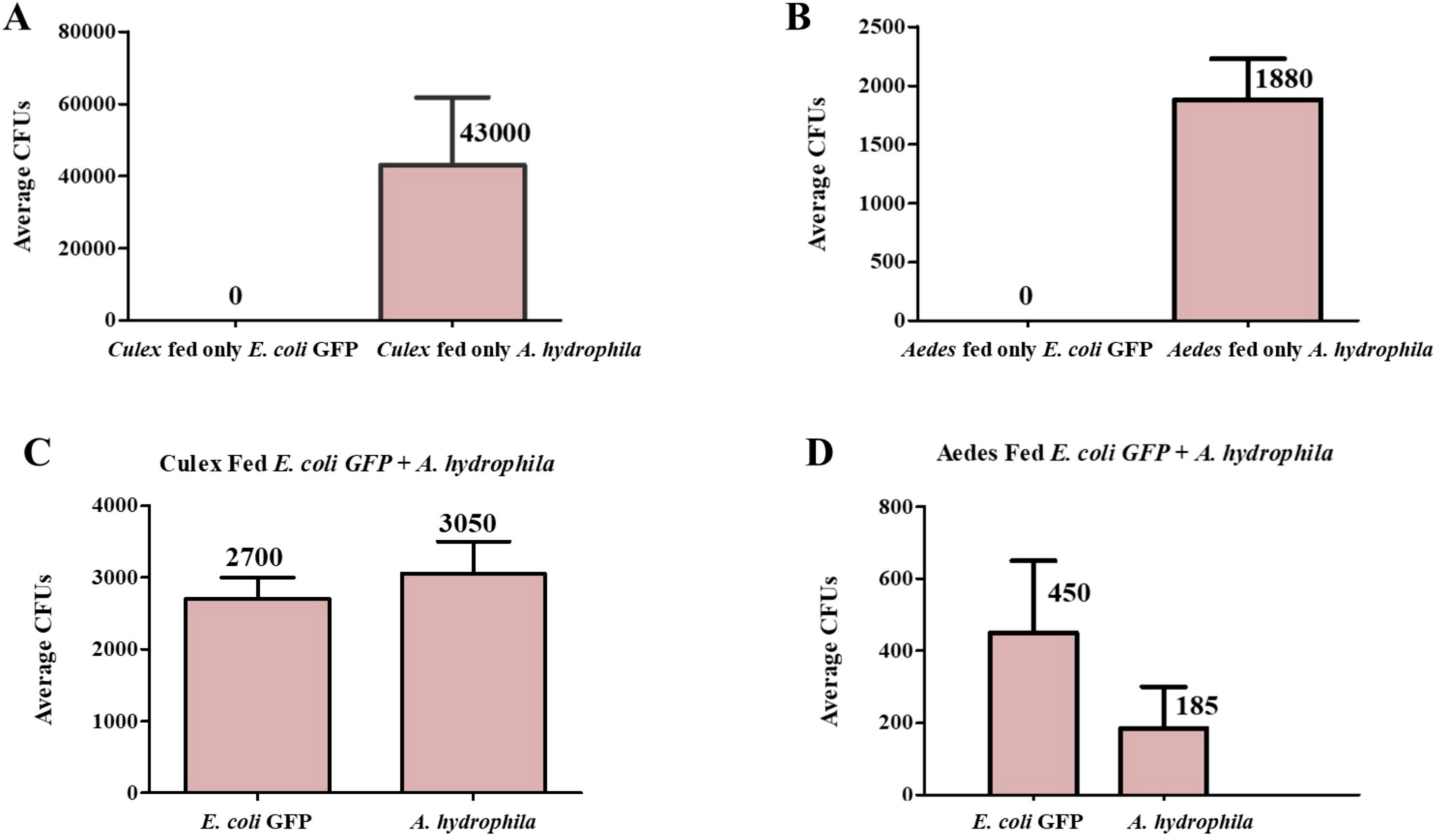
Ingestion of *A. hydrophila* leads to leaky guts in both *C. pipiens* and *A. albopictus*. Panels **(A–D)** show the calculated average number of CFUs of mosquitoes fed either *E. coli* only, *A. hydrophila* only, or *E. coli* and *A. hydrophila*. When *A. hydrophila* (OD600 = 15) was ingested by both mosquito species, CFUs were detected in the hemolymph 24 hours post feeding on the contrary to mosquitoes fed on *E. coli* (OD600 = 50) controls where no bacteria were detected in the hemolymph. When *C. pipiens* and *A. albopictus* were co-fed *A. hydrophila* and *E. coli*, CFUs of both bacteria were detected in the hemolymph.

To further validate that *A. hydrophila* inflicts gut damage, female mosquitoes were allowed to feed on sucrose supplemented with either ampicillin-resistant, GFP labelled, *E. coli* or *A. hydrophila*, for 24 hours. Bacterial suspensions were replaced by sucrose solutions for another 24 hours to ensure that no bacteria were left in the proboscis of the mosquitoes. Then, hemolymph was collected from anesthetized mosquitoes and dilutions were plated on LB plates supplemented with ampicillin. No CFUs were detected in the hemolymph when *E. coli* was administrated to the two mosquito species. In contrast, in *A. hydrophila* fed mosquitoes, high numbers of CFUs were present in the hemolymph (**Figures 3A, B**; **Supplementary Figure 1**). This indicates that *A. hydrophila* was able to cross the gut barrier, resulting in leaky gut syndrome. Finally, when *C. pipiens* and *A. albopictus* were co-fed both bacteria simultaneously, *E. coli* was detected in the hemolymph of both mosquito species (**Figures 3C, D**). This finding provides further evidence that *A. hydrophila* disrupts gut integrity, allowing a non-pathogenic bacterium to reach the hemolymph.

### Ingestion of *A. hydrophila* induces local AMPs production in the guts of *C. pipiens* and *A. albopictus*

**Figure 4:**
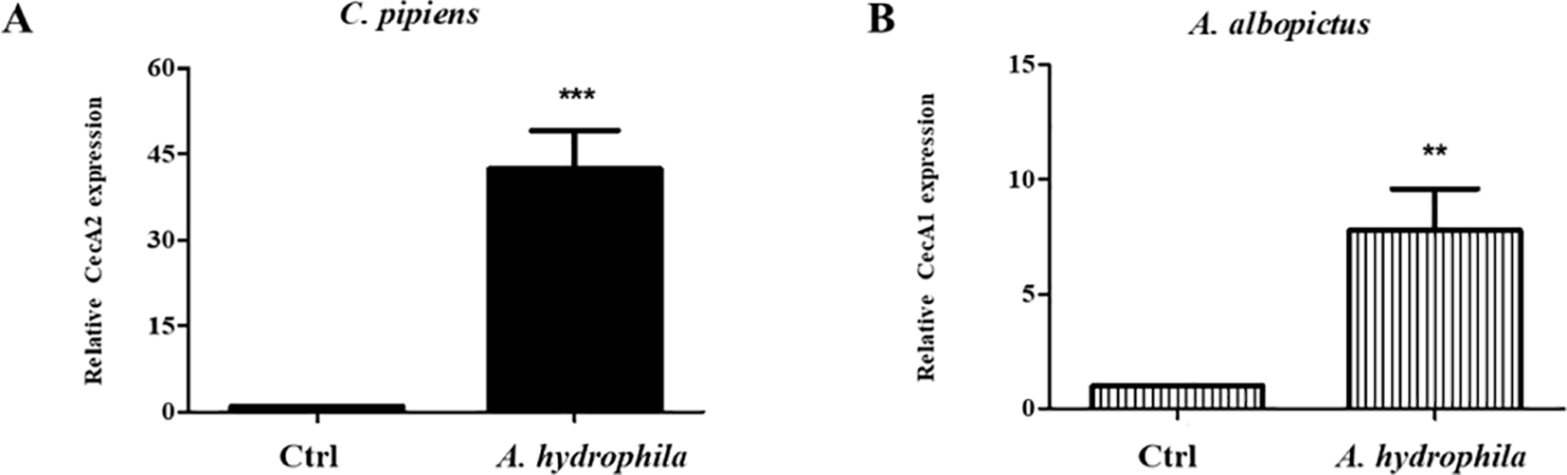
Ingestion of *A. hydrophila* induces local AMPs production in the guts of *C. pipiens* and *A. albopictus*. Relative expression levels of AMPs in *C. pipiens* and *A. albopictus* dissected guts were assayed by real time qRT-PCR 24 hours after feeding either on sucrose solutions (controls) or sucrose supplemented with *A. hydrophila*. A significant increase in the transcription levels of CecA2 and CecA1 was observed in *C. pipiens* **(A)** and *A. albopictus* **(B)** respectively in comparison to the non-infected controls (p < 0.05). Experiments were performed with a minimum of 3 biological replicates with 20 guts each and mean ± SEM were shown.

Given the pathogenicity of *A. hydrophila* and its damaging effects on the mosquito’s guts, we checked whether the ingestion of these bacteria triggers the production of local AMPs in the guts of both species. For this reason, mosquito guts were dissected 24 hours after feeding on either sucrose alone or sucrose supplemented with *A. hydrophila*. Then, qRT-PCR was performed to assess the levels of Cecropin A1 and Cecropin A2, for *A. albopictus* and *C. pipiens*, respectively. The results showed that Cecropin genes were upregulated in both mosquito guts. CecA2 levels in *C. pipiens* were approximately 42 folds higher than in the non-infected control guts (**Figure 4A**). For *A. albopictus*, CecA1 levels were approximately 8 folds higher compared to the control guts (**Figure 4B**). This proves that the mosquito’s immune system is locally triggered by *A. hydrophila*.

### *A. hydrophila* triggers the production of AMPs in *D. melanogaster* via the activation of the Imd pathway

**Figure 5:**
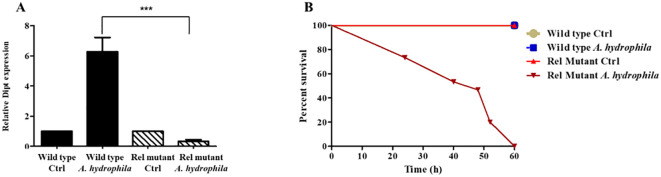
*A. hydrophila* triggers the production of AMPs via the Imd pathway and is highly pathogenic to Rel mutant *D. melanogaster*. Relative expression levels of the AMP Diptericin (Dipt) in the guts of wild-type and the Imd pathway mutant Relish (Rel^E20^) *D. melanogaster* 24 hours after feeding either on sucrose solutions (controls) or sucrose supplemented with *A. hydrophila* (OD600 = 15) **(A)**. Real time qRT-PCR shows a significant increase in the levels of Dipt transcripts in wild-type infected flies in comparison to the non-infected control group (p < 0.05). However, no upregulation of Dipt transcription was detected in the guts of Rel mutants fed on *A. hydrophila*. Survival of wild-type and Rel mutants after feeding on either sucrose solutions (controls) or sucrose supplemented with *A. hydrophila* (OD600 = 15) **(B)**. *A. hydrophila* causes significant death in *Rel* mutants while it causes no death in wild-type flies. Survivals were plotted as function of time. All survival curves had statistically significant differences (p < 0.001). The experiments were done in triplicate with 15 flies of each strain per experiment and one representative graph is shown.

Typically, Gram-negative bacterial infections induce the production of several mosquito AMPs via the Imd pathway (Zhang et al., 2017). Since *A. hydrophila* is a Gram-negative bacterium that promotes the upregulation of AMPs in mosquitoes (**Figure 4**), we speculated that this induction is mainly controlled by the Imd pathway. Taking advantage of the availability of genetic tools and mutant strains in *D. melanogaster*, we wanted to test this hypothesis. For this purpose, *D. melanogaster* flies were fed on sucrose supplemented with *A. hydrophila*, and qRT-PCR results showed a significant increase in the levels of Diptericin transcripts in wild-type infected flies, as compared to the non-infected control group. However, feeding Rel mutant flies on *A. hydrophila* did not trigger any upregulation of Diptericin transcription in the guts (**Figure 5A**). These results highlight the importance of the Imd pathway in the gut immune response to *A. hydrophila* and confirm that AMPs are locally induced in the guts in an Imd-dependent manner.

In parallel, we checked whether feeding on *A. hydrophila* kills wild-type and Imd pathway mutant *D. melanogaster*. Wild-type and Rel mutant females were fed on either sucrose alone (control), or sucrose supplemented with *A. hydrophila*, and their survival was monitored. *A. hydrophila* caused the death of Rel mutants but had no killing effect on wild-type flies (**Figure 5B**). The fact that *A. hydrophila* exerts a killing effect solely on Imd mutant flies confirms that the Imd/Relish pathway is required for *D. melanogaster* survival to this bacterial infection.

### Whole genome sequencing of the isolated *Aeromonas hydrophila* strain

**Figure 6:**
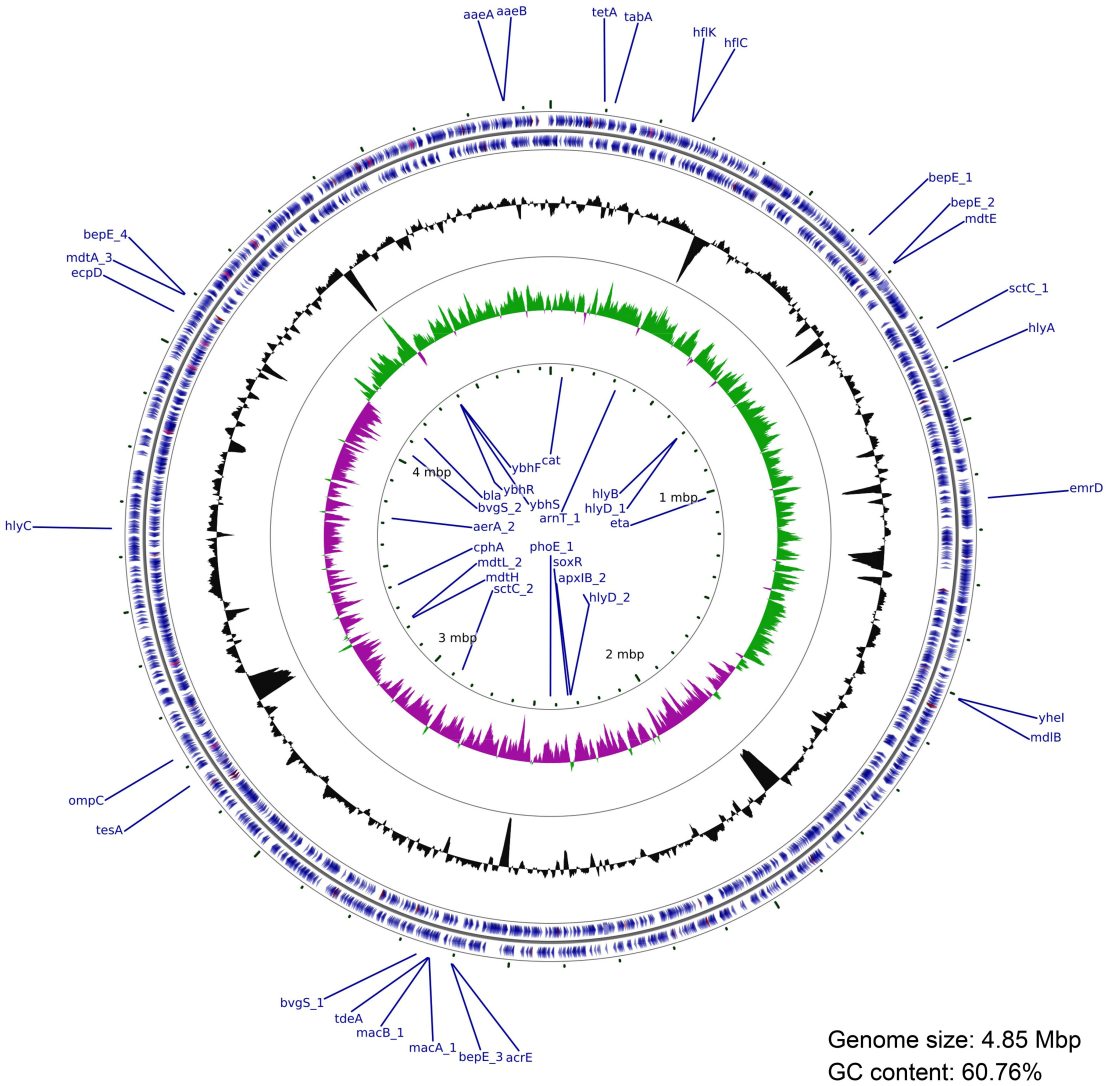
Circular genome map of the isolated *A. hydrophila.* The outer two blue rings represent protein-coding sequences (CDSs) on the forward and reverse strands, with the main virulence and antibiotic resistance genes annotated. The next ring shows GC content (black). The inner green and purple histograms represent the GC skew (green: positive; purple: negative). The central scale denotes genome coordinates in megabase (Mb). Circular genome visualization was created using the Circos package and plotted using cgview tool.

In order to gain insights into the possible mechanisms by which the isolated *A. hydrophila* strain exerts its pathogenicity, we decided to perform complete genome sequencing. The data showed that this strain possesses a repertoire of key virulence-encoding genes, such as the cytotoxic/enterotoxic genes hemolysins, aerolysin, toxin RTX-I, and exotoxin A (**Table 1**; **Figure 6**). In addition to toxin-encoding genes, type 3 secretion system (T3SS) secretin and a gene encoding chitoporin - a protein facilitating the breakdown of chitin (Suginta et al., 2013) - were also found in the *A. hydrophila* genome. Moreover, sequencing revealed a large number of antibiotic resistance genes, including β-lactamase genes (blaOXA-18, beta-lactamase, and a metallo-β-lactamase type 2), tetracycline and phenicols resistance genes, and several multidrug efflux pump genes (mdtA, mdtL etc.) (**Table 1**; **Figure 6**). Other key genes involved in pathogenicity and antibiotic resistance are listed in (**Table 1**). Altogether, these findings support the hypothesis that the isolated *A. hydrophila* strain is a mosquito pathogen, while highlighting its multidrug resistance potential.

## Discussion

Mosquitoes are among the deadliest vectors capable of transmitting infectious diseases to humans (Obradovic et al., 2022). In mosquitoes, as in other animals, the gut microbiota is a central regulator of many aspects of the host’s physiology (Liu et al., 2024). An unanswered question in microbiota research is whether the gut flora is, in its majority, transient or resident. In fact, some studies have reported the presence of core community bacterial members inhabiting the mosquito’s gut, including *Serratia* sp. and *Aeromonas* sp (Dong et al., 2009; Osei-Poku et al., 2012). Other studies argue that gut bacterial composition and diversity vary among mosquito species, but also within the same species, due to many factors, including environmental parameters and diet (Boissiere et al., 2012; Kim et al., 2015; Coon et al., 2016). In addition, most studies focusing on mosquito microbiota remain descriptive and rarely address the effects of individual bacteria on host physiology. In this regard, our work offers new perspectives on how *A. hydrophila —* a bacterium that was accidentally introduced into the insectary and capable of colonizing the gut *—* possesses detrimental effects on disease vectors, such as *C. pipiens* and *A. albopictus*.

We showed that feeding on *A. hydrophila* at OD600 = 15 kills both *C. pipiens* and *A. albopictus*. This finding suggests that, at sufficient concentrations, *A. hydrophila* acts as a virulent pathogen capable of overcoming the mosquito’s physical and immune defenses, leading to its death. Similar results were obtained in adult *D. melanogaster*, where feeding on high concentrations of *Pseudomonas entomophila* induces complete mortality within 1–2 days post infection. Nonetheless, it is important to note that the bacterial load used in our study was much lower than that used in (Vodovar et al., 2005), where they exposed flies to approximately 8x10^9^ CFU (roughly corresponding to OD600 = 100). The newly identified strain of *A. hydrophila* –which was probably introduced into the insectary via contaminated water and field-collected larvae *—* was able to colonize the guts of laboratory reared *C. pipiens* mosquitoes. This could be due to, among other factors, the low diversity of the microflora of insects kept in the laboratory under artificial conditions (Brown et al., 2023).

*A. hydrophila* has also been detected in several mosquito microbiota-profiling studies (Silva et al., 2021; Darbandsari et al., 2025; Rodpai et al., 2023). This is expected because this bacterial species is commonly found in fresh and brackish water. Moreover, a recent study by (Li et al., 2024) showed that *A. hydrophila* was the dominant bacterial species in the gut of a deltamethrin-resistant strain of *C. pipiens pallens*, and that its abundance contributes directly to the insecticide resistance. These findings emphasize the importance of specific strains in host-microbe interactions, and highlight how different strains of the same bacterium may have variable effects on the host. Future research using a lower bacterial concentration of *A. hydrophila* in different mosquito species could help explain whether the pathogenicity of the isolated bacterial strain is directly linked to its concentration, its natural characteristic, its regulation by host physiology and microbiota, or a combination of all these factors.

*A. hydrophila* was able to inflict gut damage in both *C. pipiens* and *A. albopictus* following oral feeding. This was illustrated by two main observations: 1) bacteria cross the gut barrier and enter the hemolymph (“leaky gut” syndrome), and 2) a significant increase in the number of proliferating cells in the midguts, presumably induced to replace damaged epithelial cells. Our observations align with those of (Janeh et al., 2017, 2019), which showed that the number of dividing cells in the midguts of *C. pipiens* and *A. albopictus*, increases following chemical damage or pathogen feeding. Interestingly, the number of cells in the midguts of *A. albopictus* after feeding on *A. hydrophila* is comparable to that induced by chemical damage (SDS). It is also similar to the biological damage caused by higher loads of *S. marcescens* and *Erwinia carotovora carotovora 15* (*Ecc15*) (OD600 = 50). The observed “leaky guts” phenotype indicates that the bacteria breached the gut epithelium and reached the hemocoel, which reflects a critical failure in the host’s immune defenses, notably in the gut barrier. A similar result was observed in *D. melanogaster*, where feeding on *S. marcescens* led to leaky guts, and ultimately the death of the flies 6 days post infection (Nehme et al., 2007). It is known that gut homeostasis is maintained by a balance between cell damage caused by bacterial infections, and epithelial repair by stem cell division (Buchon et al., 2009). In *A. hydrophila* infection, the balance skewed towards gut damage and mortality. Regardless, this work reinforces the importance of maintaining gut integrity as a defense mechanism, which increases the tolerance of mosquitoes to infections.

In mosquitoes, as well as in *Drosophila*, Gram negative bacteria activate the Imd signaling pathway leading to the production of local AMPs (cecropins, diptericin) in the gut (Buchon et al., 2014). Here, we show that, upon ingestion of *A. hydrophila*, local immune responses are induced in the guts of both *C. pipiens* and *A. albopictus* mosquitoes. This is in agreement with (Janeh et al., 2017), who showed that feeding on *S. marcescens* induces a slight but significant increase in CecA1 levels in the guts of *A. albopictus* mosquitoes. Nevertheless, the local immune response induced by the *A. hydrophila* strain isolated in the present study was stronger in both *C. pipiens* and *A. albopictus* guts, suggesting that this AMP upregulation can be aggravating gut damage (Broderick, 2016). On another note, in the model organism *Drosophila*, Rel mutant flies failed to induce AMPs and succumbed to *A. hydrophila* feeding suggesting an efficient role of the Imd pathway in fighting this bacterium.

Finally, whole genome sequencing of this pathogenic *A. hydrophila* strain showed that it contains an array of virulence and antibiotic resistance genes. For example, the presence of cytotoxic genes (aerolysin) indicates that this strain is able to induce cell lysis via pore formation (Abrami et al., 2003). Also, it has been shown that hemolysin (another gene found in this *A. hydrophila* isolate) possesses enterotoxic activity, which can disrupt intestinal cells (Fujii et al., 2003). This hints at possible mechanisms through which *A. hydrophila* damages the mosquito gut. Moreover, one notable virulence factor found in this strain, is the T3SS secretin, which is used to inject effector proteins into host cells (Coburn et al., 2007), and is known to confer pathogenic Gram-negative bacteria, including *Aeromonas* sp. unique virulence mechanisms (Frey and Origgi, 2016). The presence of a gene encoding chitoporin was also significant, since it indicates an ecological adaptation to insect hosts (chitin-rich environment). On the other hand, the high abundance of antibiotic resistance genes suggests that this strain of *A. hydrophila* has been exposed to many antimicrobial agents in its environment, and could constitute a threat to human health. Altogether, this work adds a new piece to the puzzle of the tripartite host-microbiota-pathogen interactions, with possible implications for microbiota-based vector control strategies.

## Data Availability

The datasets presented in this study can be found in online repositories. The names of the repository/repositories and accession number(s) can be found below: https://www.ncbi.nlm.nih.gov/, SRS25118285.

## Supplementary material

Supplementary Figure S1 Feeding on SDS significantly increases the number of mitotic cells in the midguts of *C. pipiens* and *A. albopictus*. Quantification of these results show that the increase in the number of replicative cells at the level of the midguts is significant in both mosquito species (p < 0.0001). In SDS fed *C. pipiens* mosquitoes **(A)**, an average of 15.60 ± 1.585 positive cell per midgut (n=15) was observed as compared to the midguts of sucrose fed mosquito that showed an average of 2.200 ± 0.3117 (n=15). *A. albopictus* mosquitoes exhibited a similar response **(B)** 10.07 ± 0.7589 dividing cell per midgut (n=15) was detected when compared to controls that had 1.267 ± 0.2482 dividing cell per midgut (n=15).

Supplementary Figure S2 Ingestion of *A. hydrophila* leads to leaky guts in both *C. pipiens* and *A. albopictus*. When *A. hydrophila* (OD600=15) was ingested by both mosquito species, CFUs were detected in the hemolymph 24 hours post feeding **(A–D)** on the contrary to mosquitoes fed on *E. coli* (OD600=50) controls where no bacteria were detected in the hemolymph **(B–E)**. When *C. pipiens* and *A. albopictus* were co-fed *A. hydrophila* and *E. coli*, CFUs of both bacteria were detected in the hemolymph **(C–F)**.
