# Supplementary figures and images for: Identification of an Aeromonas hydrophila strain as a new mosquito pathogen
Source: Front Cell Infect Microbiol. 2025 Aug 12;15:1649545. doi: 10.3389/fcimb.2025.1649545 (PMC12378139; doi:10.3389/fcimb.2025.1649545)

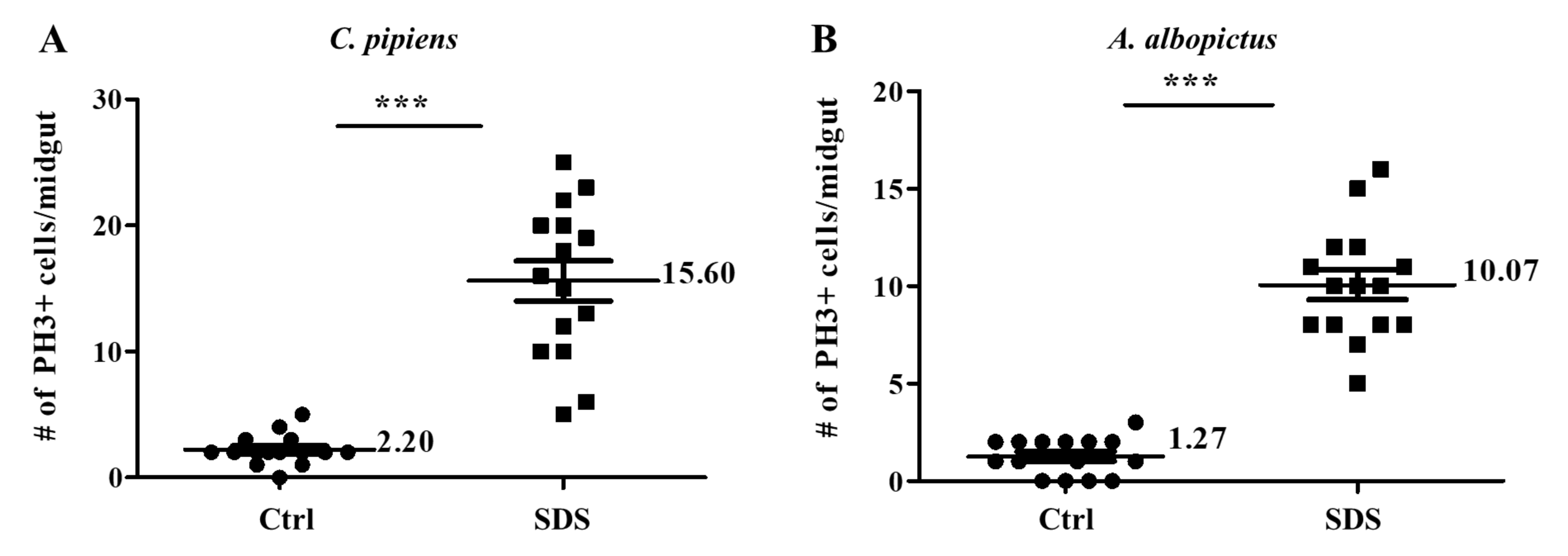

Supplement: Supplementary Figure S1 — Feeding on SDS significantly increases the number of mitotic cells in the midguts of C. pipiens and A. albopictus. Quantification of these results show that the increase in the number of replicative cells at the level of the midguts is significant in both mosquito species (p < 0.0001). In SDS fed C. pipiens mosquitoes (A), an average of 15.60 ± 1.585 positive cell per midgut (n=15) was observed as compared to the midguts of sucrose fed mosquito that showed an average of 2.200 ± 0.3117 (n=15). A. albopictus mosquitoes exhibited a similar response (B) 10.07 ± 0.7589 dividing cell per midgut (n=15) was detected when compared to controls that had 1.267 ± 0.2482 dividing cell per midgut (n=15). [file Image1.tiff]

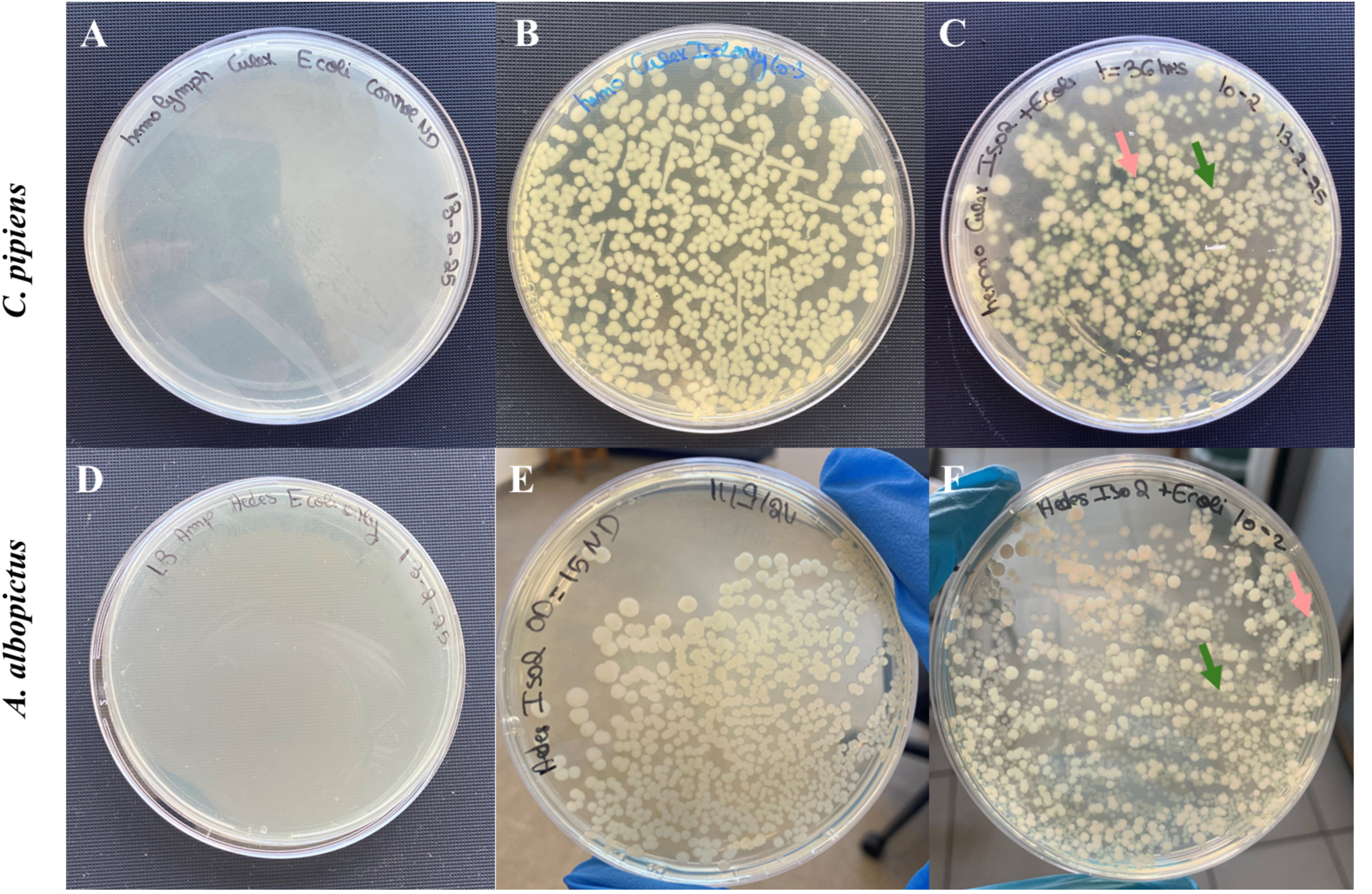

Supplement: Supplementary Figure S2 — Ingestion of A. hydrophila leads to leaky guts in both C. pipiens and A. albopictus. When A. hydrophila (OD600=15) was ingested by both mosquito species, CFUs were detected in the hemolymph 24 hours post feeding (A–D) on the contrary to mosquitoes fed on E. coli (OD600=50) controls where no bacteria were detected in the hemolymph (B–E). When C. pipiens and A. albopictus were co-fed A. hydrophila and E. coli, CFUs of both bacteria were detected in the hemolymph (C–F). [file Image2.tiff]
